# Barriers and facilitators for implementation of HPV-based cervical cancer screening in Tanzania: a qualitative study among healthcare providers, stakeholders, and Tanzanian women

**DOI:** 10.1080/16549716.2025.2491852

**Published:** 2025-04-24

**Authors:** Karen Grønlund Madsen, Julie Skipper Mosgaard, Martha Oshosen, Patricia Swai, Julius Mwaiselage, Vibeke Rasch, Ditte Søndergaard Linde

**Affiliations:** a Department of Obstetrics and Gynaecology, Odense University Hospital, Odense, Denmark; b Department of Clinical Research, University of Southern Denmark, Odense, Denmark; c Kilimanjaro Clinical Research Institute, Kilimanjaro Christian Medical Centre, Moshi, Tanzania; d Department of Obstetrics and Gynaecology, Kilimanjaro Christian Medical Centre and Kilimanjaro Christian Medical University College, Moshi, Tanzania; e Department of Cancer Prevention Services, Ocean Road Cancer Institute, Dar es Salaam, Tanzania

**Keywords:** Cancer, HPV, WHO, the social ecological model, East Africa, prevention, preventive medicine, interview

## Abstract

**Background:**

Cervical cancer is the leading cause of cancer-related deaths in Tanzania and the most common form of cancer among Tanzanian women. Screening attendance remains among the lowest globally, necessitating improved attendance and screening methods.

**Objective:**

This study aims to assess the feasibility of implementing the World Health Organization’s 2021 hPV-based screening guideline in Tanzania by identifying potential barriers and facilitators to HPV-based screening among screening clients, healthcare providers, and stakeholders.

**Methods:**

From October 2022 to February 2023, 25 semi-structured interviews were conducted with screening clients (*n* = 16) and healthcare providers and stakeholders (*n* = 9) in Moshi and Dar es Salaam. Data were analyzed using a deductive framework based on Bronfenbrenner’s Social Ecological Model, supplemented with inductive subcategories from the transcripts.

**Results:**

Barriers and facilitators emerged across all levels of the Social Ecological Model. At the individual level, clinic-based screening and a one-visit approach were barriers, while HPV-self-sampling was a facilitator. Interpersonal barriers included limited social support, while referrals served as facilitators. Community-level barriers included fear and misconceptions, countered by facilitators such as increased awareness and health education. Health system challenges included restrictive age limits and urbanization of human resources, with uptake through other health services acted as a facilitator. Political barriers highlighted the need for a steady local supply chain, while cost reduction could serve as a facilitator for guideline implementation.

**Conclusion:**

WHO’s 2021 hPV-based screening guideline shows promise in Tanzania, but barriers such as clinic availability, fear, misconceptions, and supply chain issues must be addressed to ensure successful implementation.

## PAPER CONTEXT

- **Main findings:** Barriers and facilitators for implementing the 2021 World Health Organization guideline on human papillomavirus-based cervical cancer screening in Tanzania were identified across individual, interpersonal, community, health system, and political levels.
- **Added knowledge:** The study highlights the need for clinic-based screening, a one-visit approach, and strategies to address fear, misconceptions, and gaps in supply chains to enhance human papillomavirus-based screening programmes in low-resource settings.
- **Global health impact for policy and action:** Implementing human papillomavirus-based screening with tailored strategies can reduce cervical cancer disparities and improve health outcomes in low- and middle-income countries.

## Background

In 2020, an estimated 342,000 women died from cervical cancer and 604,000 women were diagnosed with the disease, making cervical cancer the fourth most common form of cancer among women globally [1]. In many low- and middle-income countries (LMIC), cervical cancer is the leading form of cancer and responsible for most cancer-related deaths [2]. The global disparity in cervical cancer is evident, and studies have shown that incidence and mortality are inversely proportional to the mean national human development index (HDI), i.e. the incidence is three times higher and mortality six times higher in low-HDI countries compared to high-HDI countries [3]. The largest burden of cervical cancer is found in East Africa, with a global age standardised incidence rate (ASIR) of 40.1 per 100.000 women-years compared to a world average of 13.3 ASIR per 100.000 women-years [3]. Tanzania is one of the countries in the world with the highest incidence rate of cervical cancer, with an ASIR of 62.5 per 100.000 women-years, and the disease is the most common form of cancer in the country and the leading cause of cancer deaths among Tanzanian women [4].

Persistent infection with a high-risk (HR) type of human papillomavirus (HPV) is the primary cause of cervical cancer, and effective primary (HPV vaccination) and secondary prevention (screening for and treatment of precancerous lesions) will prevent most cervical cancer cases [5,6]. Studies have shown that the high rates of cervical cancer incidence and mortality in sub-Saharan Africa are mainly attributable to insufficient HPV-vaccination rollout, lack of high-quality cervical cancer screening and lack of widespread high-quality treatment [5,7,8]. Currently, HPV vaccination is being rolled out in Tanzania, targeting adolescent girls [9,10]. However, systematic and effective screening has proven to be challenging, and Tanzania has one of the lowest uptakes of cervical screening in the world (approximately 13%) [10]. To overcome the immense burden of disease in the country, in addition to vaccination campaigns it is necessary to also improve the current screening programme to reach the women who have not been vaccinated [11].

In 2021, the World Health Organization (WHO) announced a new guideline for screening and treatment of cervical pre-cancerous lesions in high-income countries (HIC) and LMIC [12]. The guideline strongly depends on HPV detection as the new primary screening tool and suggests that all women over the age of 30 must have an HPV detection test in either a screen-and-treat or a screen, triage, and treat approach every 5–10 years. Women living with HIV (WLWH) have increased risk of contracting the disease and should therefore begin screening at the age of 25 and be tested more frequently (every 3 to 5 years) in a screen, triage, and treat approach according to the guideline [12].

Screen-and-treat with HPV detection is not yet available in many LMIC countries, including Tanzania [13]. Consequently, positive screening results are being followed up over long periods of time and through various triage strategies depending on what is available in the individual countries, which increases the risk of lacking follow-up. The possibility of self-sampling with HPV-based screening can provide the opportunity to test in one’s own home or at a clinic nearby, thereby reducing time spent on clinic visits, traveling costs, and lacking follow-up. Additionally, HPV detection tests have proven to be cost effective and sensitive to women’s comfort and privacy, making them useful in sub-Saharan Africa [14–17]. Therefore, HPV self-sampling is considered a screening method that may improve screening attendance.

If Tanzania is to follow the latest WHO guideline for cervical screening, it will involve switching from the current screen-and-treat/single-visit approach, which involves visual inspection of the cervix with acetic acid (VIA) or Lugol’s iodine combined with either cryotherapy, cold or LEEP treatment [18]. As HPV-based screening is not yet available in the country, implementation of HPV-based screening in Tanzania is facing multiple unaddressed challenges, i.e. expenses linked to supplies and education of healthcare professionals, longer waiting time for results with risk of lost to follow up, women and healthcare provider’s knowledge about HPV, and especially, little experience with actual implementation of HPV-based screening in sub-Saharan African countries.

## Methods

### Aim

The overall aim of this qualitative study was to assess the feasibility of implementing the World Health Organization’s 2021 guideline for cervical cancer screening in Tanzania. Our specific objective was to understand barriers and facilitators for an HPV-based screening programme in Tanzania according to Tanzanian women who have participated in both VIA- and HPV-based screening, and to Tanzanian healthcare workers and stakeholders working with the Tanzanian cervical cancer screening programme.

### Study context and setting

In 2015, the Comprehensive Cervical Cancer Prevention in Tanzania (CONCEPT) project was established as a cooperation between Ocean Road Cancer Institute (ORCI) in Dar es Salaam, Kilimanjaro Christian Medical Centre (KCMC) in Moshi, the University of Southern Denmark (SDU), and the Danish Cancer Society’s Research Centre. The aim of CONCEPT was to substantially improve cervical cancer prevention in Tanzania and thereby reduce the burden of disease due to cervical cancer. A cohort of 4080 Tanzanian women between the age of 25 and 60 with an oversampling of WLWH, was established by enrolment through three cervical cancer screening clinics in Dar es Salaam and the Kilimanjaro Region. Women were excluded if they were pregnant, had a recent history of premalignant lesions, had previously been diagnosed with cervical cancer, or had ever undergone abdominal hysterectomy [19]. Among other things, the CONCEPT study investigated the contraction and persistence of HR HPV and associated risk factors, the feasibility and acceptability of HPV self-sampling, and how to increase follow-up on HPV-positive women [19]. This qualitative study is a sub-study in the CONCEPT cohort and was conducted at KCMC and ORCI between October 2022 and February 2023.

### Participants

#### Cervical cancer screening clients

Women were eligible for this study if they were part of the CONCEPT cohort and had experience with VIA-based screening (standard care) and HPV-based screening through both provider-collected screening and self-sampling. No further inclusion or exclusion criteria were established. Women who had previously tested HPV-positive as part of the CONCEPT study were scheduled for a re-examination at KCMC, where they collected an HPV self-sample kit and underwent a gynaecological examination. After this re-examination, the women were invited to participate in this qualitative study.

#### Healthcare providers and stakeholders

The healthcare providers and stakeholders, including a senior person at ORCI and a representative from WHO in Tanzania who participated in the study, were all included based on their expertise and experience within cervical cancer, HPV screening, and the Tanzanian screening programme. Some of the participants had previously worked with the CONCEPT project and were therefore familiar with the overall purpose.

### Data collection

Semi-structured interviews with the screening clients were conducted in Kiswahili with simultaneous English translation. A female Tanzanian social scientist, who had experience with qualitative interviews, served as translator. She was briefed about the study objectives and the interview guide prior to the interviews. The first author interviewed all participants while the second author took notes. After the first four interviews, the interview guide was reviewed by the first and second author and the translator, and small changes were made. Data collection stopped when saturation was reached, defined as the point when no new information arose from the interviews. *An estimate before start was that we would possibly need a sample of 12 to 20 participants to reach saturation of the material*. The interviews took place at KCMC in a private room at the Reproductive Health Centre in the presence of the first and second authors, the translator, and the interviewee. Prior to the interview, all women were informed about the overall aim of the study and introduced to the consent form. If the women chose to sign the consent form, the interviews began immediately after. All interviews were audio-recorded in full length with simultaneous translation for further analysis, and *all* participants received a modest compensation in appreciation of their time.

Interviews with healthcare providers and stakeholders were conducted in English by the first author, with one exception: one interview was conducted in Kiswahili with simultaneous English translation from the same translator who conducted the translation of the remaining interviews with the women. The interviews took place at either ORCI or KCMC according to the healthcare providers’ workplace, or were conducted online over Zoom. All interviews were audio-recorded in full length, and Zoom interviews were also video-recorded.

### Interview guide

Semi-structured interviews were conducted with all participants by use of an interview guide. The interview guide for screening clients consisted of 4 key questions [see Supplementary file 1] and was inspired by previous qualitative studies regarding HPV-based screening conducted during the CONCEPT project [15,20,21]. The interview guide contained questions concerning the screening experience at the re-examination prior to the interview, screening with the HPV self-sample test, screening with VIA, comparison of the VIA and HPV test, and the general screening programme in Tanzania. It also contained a brief health education section about the differences- and pros and cons of the two tests to investigate if more information and education changed the women’s view on the screening methods. The interview guide for the healthcare workers and stakeholders consisted of 5 key questions [see Supplementary file 2] and was based on selected key themes from the WHO guidelines for cervical cancer screening [12]. It consisted of questions regarding the current screening programme with VIA, the new 2021 guidelines from WHO, execution of screening, economy, laboratory facilities, stock issues, and personnel challenges.

### Data analysis

All interviews were initially transcribed from audio files using transcription software from Otter.ai [22], and subsequently manually verified by the first and second authors. Data were analysed using NVivo 12 [23] by use of combined deductive-inductive coding. Central themes were deducted using a coding frame based on Bronfenbrenner’s Social Ecological Model [24], which has been used as an analytical framework for multiple cervical cancer studies [25–28]. The model describes five levels at which cervical cancer screening can be analysed: 1) Individual, 2) Interpersonal, 3) Community, 4) Health Systems, and 5) Policy [24]. The model guided the transparency of the complexity of introducing new screening methods in a country, as the different levels are often intertwined and inseparable. The coding frame was supplemented with subcategories that inductively arose from the transcript [see Supplementary file 3]. All transcripts were analysed and coded by the main author, and to test the reliability of the coding, the second author recoded 20% of the transcripts and the last author revised and commented on the coding process. This led to minor changes in the coding frame, and therefore the data were revisited and recoded for a second time by the first author. Prior to the transcription, all women’s IDs were replaced by the top 16 Tanzanian feminine names [29] to de-identify the women, and ‘she’ was replaced with ‘I’ in the transcripts for a first-hand account. All socio-demographic characteristics required were derived from the CONCEPT database.

## Findings

### Characteristics of study participants

A total of 16 screening clients and nine healthcare providers or stakeholders participated in the study. Seventeen screening clients were invited to participate in the study; one screening client declined due to time constraints. The mean age of the screening clients was 49.8, and the majority had attended primary school (88%, n = 14/16). Further, 69% (n = 11/16) of the women were living with HIV (Table 1). All interviews were conducted between 9 a.m. and 5 p.m. with a mean duration of 33 mins. [22–51 min.].

**Table 1.** Characteristics of screening clients participating in interviews regarding cervical cancer screening (N = 16).

Characteristics (N = 16)	n (%)
**Age, mean (SD)**	49.8 (7)
**Age groups**	
<40	1 (6)
40–50	8 (50)
50–60	5 (31)
>60	2 (13)
**Educational level**	
≤ Primary (standard 5–7)	14 (88)
≥ Secondary (form 1–4)	2 (13)
**Marital status**	
Married, monogamous	7 (44)
Single, with regular partner	2 (13)
Divorced/widow	7 (44)
**Parity**	
Never pregnant	15 (94)
1–2	1 (6)
**Religion**	
Christian	11 (69)
Muslim	5 (31)
**HIV status**	
Positive	11 (69)
Negative	5 (31)

A total of eight healthcare providers and two stakeholders were invited to participate in the semi structured interviews; nine agreed to participate. Three of the interviews were conducted via Zoom [30]. Two nurses and five doctors from either KCMC or ORCI, a senior person at ORCI, and a representative from WHO in Tanzania participated in the study. Average years of experience with cervical cancer screening were 9.7 years [3–18 years] (Table 2). Five out of eight healthcare providers also had extensive research experience with the subject of HPV and cervical cancer in addition to their clinical experience. The mean duration of the interviews with healthcare providers and stakeholders was 37 mins. [21–61 min].

**Table 2.** Characteristics of Tanzanian healthcare professionals participating in interviews regarding cervical cancer screening (*N* = 9).

Interviewees	n	Gender		Years of experience with cervical cancer screening			
		Female	Male	0–5	6–10	11–15	> 15
Nurse	2	2	–	1	-	–	1
Medical doctor	5	–	5	1	2	2	–
Senior person at ORCI	1	–	1	–	–	–	1
WHO representative	1	–	1	1	–	–	–
Total	N = 9	2	7	3	2	2	2

**Figure 1.**
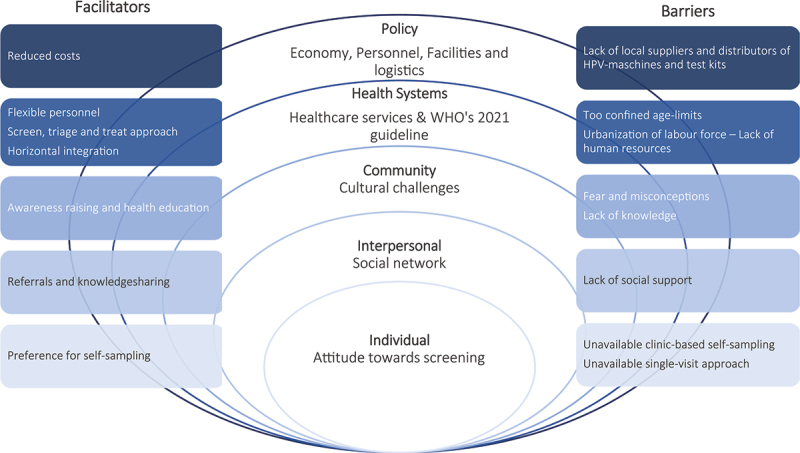
The socio-ecological model applied to HPV-based cervical cancer screening showing barriers and facilitators.

In addition to the five main levels of the Socio-Ecological Model, multiple subcategories arose under each level (Figure 1), which are elaborated in the following sections.

### Individual

#### Attitude towards screening

A benefit of HPV-based screening was that HPV-based self-sampling was associated with less pain and discomfort compared to VIA-based screening, which entails a gynaecological examination. For example, as Mary explained her self-sampling experience: *‘I was anticipating some kind of pain, but when I was instructed and I successfully inserted it, I did not feel anything’* (Mary).

HPV-based screening also allows for conducting the screening at home instead of having to come to the clinic. However, according to the screening clients, it was not feasible to conduct the screening at home. Instead, they preferred to conduct the test at the clinic. As Veronica explained:

It is much better here [ed. at the clinic]. More than [ed. at] home. Because at home you might miss some information, or you might do it wrong. […] [ed: There is] no one to tell you that you are doing it wrong. (Veronica)

Further, the women were worried about the interference of children or everyday life. For example, as Mariam said: *‘As long as you have kids in your house […] they might think […] it is a toy. […] It is not that safe at home’* (Mariam). This was backed by the healthcare providers. For example, one doctor said:

I do not think it is feasible [ed. to do the self-sample at home]. Because most of the time, […] [ed: when] it has been done, […] instead of self-sampling, it ended up with being provider-assisted. (Medical Doctor)

However, healthcare providers agreed that self-sampling had the potential to solve some of the women’s concerns about screening, i.e. lack of privacy and distance to screening services. Yet, healthcare providers pointed out that there was a need for extensive education if Tanzanian women were to be able to collect a proper quality self-sample on their own. For example, the WHO representative said: *‘There’s been an issue of environmental challenges. Maybe they won’t take the sample properly’* (WHO representative), and another doctor said:

[…] You need to have an education first for the clients. […] Where do you get the devices first? How do you take this specimen? What are the logistics in terms of where do you send a specimen for getting the results? Those logistical issues need to be understood from a client’s standpoint, before [ed: self-sampling can be] […] implemented. (Medical Doctor)

According to the screening clients, the largest barrier regarding HPV-based screening compared to VIA-based screening was having to wait for the results. The women stated that they preferred to receive the result right away as they did with VIA. However, other women also stated that they were willing to wait for the results, while one woman in contrast actually preferred waiting, because she thought it was an indication of a better test. Elizabeth, who wished to know the results right away, described the ambiguity regarding this issue:

I am not really comfortable with that period of waiting. I wish I could get the results today. But because there are so many women coming here to do the test, I have to […] accept the reality and wait for the test results. But my wish is to just get the test results soon after the screening. (Elizabeth)

This was also an issue that was brought up by the healthcare providers and stakeholders. However, according to them, the main issue with having to wait for the test results was that it would increase the risk of lacking follow-up. This tapped into the issue of not being able to perform see-and-treat as it is possible with VIA-based screening. For example, the WHO representative said:

[…] The mother is the one who looks out for the family. Spending 10 to 12 hours in the clinic, the home will be totally shut down, the kids will not eat properly. So, we need the machine, that will be give quick results within short time. (WHO representative)

### Interpersonal

#### Social support

Generally, lack of social support for screening – whether VIA-based or HPV-based screening – was perceived as a key barrier for participating in cervical cancer screening. Some women explained how they experienced opposition from close friends. For example, as Fatuma said:

Sometimes my friends, […] they are telling me not to go. […] [ed: They say] the further you know about your cervical cancer status, the more you are likely to be dead. (Fatuma)

Lack of social support could also be an underlying reason for women not wanting to conduct HPV self-sampling at home. Further, Joyce also experienced resistance when she tried encouraging her sick female relative to seek medical counselling,

Whenever I am insisting that she come and take a test, she says ‘As long as […] [ed: it] does not hurt, why should I bother?’ So, people out there are sick, terribly sick, but they do not want to […] seek […] care. So, it is a problem. (Joyce)

On the other hand, word of mouth, knowledge sharing and encouragement from woman to woman could benefit the screening programme and the attendance. As Neema, who was contacted by a nurse to attend screening, said: *‘Nurses should make more calls to invite people, and for me after I’m done with the test, I can go and tell someone’* (Reema).

### Community

#### Cultural challenges

According to the screening clients, misconceptions regarding the screening process and fear of both screening and tests results were major reasons why Tanzanian women do not attend cervical cancer screening. For example, as Mariam said about VIA and gynaecological examinations:

[…] There is this misconception about the test. […] It is like […] you take all of […] [ed: the cervix] out for the test, so they can imagine all that torture and pain. That is what is keeping them from coming. (Mariam)

Another major barrier to overcome if HPV-based screening is to be implemented in Tanzania is lack of knowledge about HPV, cervical cancer, and the epidemiology of the disease. When asked about what HPV-self-sampling tests for, none of the women were aware, and when we asked about their knowledge of what it meant to be HPV-positive, only two knew that the virus was a risk factor for developing cervical cancer. Some women mentioned this knowledge gap as a reason why women in Tanzania do not attend screening. This concern was shared by healthcare providers:

[…] Knowledge and awareness in the public about cancer and cervical cancer in particular […] [ed: are] limited. In addition […] the high agenda or priority […] [ed: is] on communicable diseases. So, cancer, being a non-communicable disease, is not on the main agenda. (Medical Doctor)

To improve screening attendance overall – and in particularly regarding HPV-based screening – increasing awareness and education was therefore considered to be crucial. As Grace said:

Education and awareness should be provided to the community, not only in the church, but also in local government meetings, where lots of people are meeting together. Or any other […] [ed: place] where there is a gathering of people, […] health education should be provided there. (Grace)

A benefit of HPV self-sampling compared to VIA-based screening was that self-sampling was considered to be more private. Both screening clients and healthcare providers mentioned having to undress for the gynaecological examination was a cultural issue to which HPV self-sampling could be a solution. Also, distance to healthcare facilities was mentioned as a potential problem, which could also be eliminated by the possibility of remote testing. For example, as Maria said:

[…] Maybe such screening service […] [ed: could] be available even in dispensaries […] [ed: local health facilities]. Because other people they might think KCMC is too far from their place, so maybe they could be willing to come because it is close to their home. (Maria)

### Health systems

#### Interaction with healthcare services

Interaction with other local healthcare services appeared to be a great resource for enrolment into the screening programme for both VIA-based and HPV-based screening. Five out of 16 women were referred through HIV care and treatment clinics (CTC), four women were referred through other local health services, and two women were contacted by healthcare providers and encouraged to join via home visits. Contrary to this, recruitment and health education through other programmes were still considered an unexploited resource among the healthcare professionals:

[…] Women who are going here for diabetes, for hypertension, and other diseases, […] should be informed […] simple questions, what is cervical cancer? What are the symptoms? What can you do to prevent it? […] So, whenever the opportunity arises, [ed: when] women they come to the hospital, maybe when they are waiting for other services, then they should be advised [ed: to get cervical screening]. (Medical Doctor)

#### The World Health Organization’s 2021 guideline

The healthcare providers and stakeholders were all asked about their professional opinion on the details of the WHO’s 2021 guideline. Regarding the age limit for screening, which is 30–50, the professionals pointed out that they thought it was too restrictive, and that the lower limit should be lowered even more. This was based on their own professional experiences with cervical cancer among women in their twenties,

They start sexual activities pretty early. Now, they contract this infection very early. So, if you wait to screen until they are 30, it could be too late, especially if they are HIV infected. (Medical Doctor)

A medical doctor also argued that there should be no upper limit for screening, because there will be women older than 50 who have never attended screening due to the low screening uptake in Tanzania, and they should never be denied screening.

When asked if Tanzania should opt for the screen-and-treat or screen, triage, and treat approach, the healthcare providers and stakeholders agreed that it was necessary to use screen, triage, and treat through VIA. This was to avoid overtreatment because of the high HIV burden in the country, which causes many women to become infected with HPV, and to optimise treatment:

[…] You will see the woman has positive results, but you do not know where in the cervix there is a lesion. […] Precancerous lesions start in a particular area, not the whole cervix. So, the issue if you want to do see and treat with HPV DNA testing, you will need to do cryo for the whole of the cervix without knowing where your target was. (Senior person at ORCI)

Hence, once a woman has tested HPV positive, she will need triaging with VIA to know whether treatment is indicated and to improve treatment when it is indicated.

### Policy

#### Economy

Overall, stakeholders and healthcare providers agreed that there was a need for cost analysis before HPV-based screening could be implemented nationally in Tanzania. To successfully work and be sustainable, healthcare providers agreed that the programme needs to be financed by the government rather than relying on NGOs and donations. It was also pointed out that HPV-based screening would be more cost-effective than VIA-based,

[…] It is cost effective to use HPV compared to VIA, because we will be able to capture a lot of early premalignant lesion in the cervix, […] and […] be able to reduce the burden of cervical cancer, and […] reduce the cost for the government. […] We will be able to have women being screened at longer duration compared to VIA. […] [Ed: This shows that] the government can actually save resources […] with HPV testing compared to VIA. (Medical Doctor)

#### Personnel

All healthcare providers and stakeholders agreed that medical personnel were flexible in regard to changing the screening protocol. No barriers were found in terms of skills if transitioning training was considered, but some did see problems regarding the number of trained personnel as well as their distribution at a national level:

We have insufficient staff at the country level. […] You [ed: will] find a clinic being run by one or two [ed. healthcare providers]. […] If this screening personnel went for the refresher training for a week or two weeks, that clinic would be closed. […] [ed: We need to] hire more and do a proper distribution. (WHO representative)

#### Facilities and logistics

The largest barrier at the policy level was a stable supply chain for HPV tests, reactants, and machines. Healthcare providers and stakeholders saw no need for expanding laboratories or testing facilities, but local manufacturers are key to achieve programme sustainability:

So, the programme, or the recommendation, is good, but the implementation has a challenge. So, one of the challenges is the machine itself. The machines for HPV DNA testing. They need to be procured to the country, to all the facilities. The second challenge […] is the reactants, they have not been available lately. We do not have local dealers who can supply the reactants. (Senior person of ORCI)

as a solution to the need for additional machines, a representative from the WHO mentioned underutilisation of existing machines that are being used in HIV and tuberculosis (TB) testing:

There has been an issue of verticalisation of programmes in Tanzania, rather than integration, so the TB they say, ‘this is our machine, you cannot use them for other screening’ […] Everyone runs vertical with their own reason, I’m not sure, […] why they want that rather than integration. So, there is an underutilisation of our machines. (WHO representative)

The Socio-Ecological Model with subcategories applied to HPV-based cervical cancer screening in Tanzania showing barriers and facilitators for each level.

## Discussion

This qualitative study shows that there are multiple barriers that need to be addressed at all societal levels for Tanzania to be able to successfully implement HPV-based screening in accordance with the WHO’s 2021 guideline for cervical cancer. Individual barriers include the inaccessibility of clinic-based screening at primary healthcare levels, and a need for a one-visit approach to HPV-based screening with same-day results. At community level, overcoming fear and misconceptions is key, there is a need for adjustment of the guidelines to fit Tanzanian needs and for a local and steady supply chain of HPV machines and reactants for test kits at health system and political level. Facilitators for implementing the guideline include the fact that HPV-based screening with self-sampling is preferred over gynaecological screening at an individual level, and increasing awareness and knowledge among the target group is possible at the community level. At the health system level, horizontal integration is an unexploited resource, personnel are considered flexible and capable, and there is a possibility of cost reductions at the political level when implementing HPV-based screening.

### Comparison with previous literature

Overall, our findings are somewhat compatible with those found in other studies conducted in sub-Saharan Africa. Lack of knowledge about HPV and HPV testing has proven to be a general problem [31,32], together with misconceptions and stigmatisation [33,34]. A qualitative study from Nigeria conducted among 61 WLWH showed that even though most of the women have heard of cervical cancer, they were not aware of their own increased risk of contracting HPV and thereby increased risk of developing cervical cancer. The study further suggested integration of cervical screening in HIV treatment facilities [34]. This has in fact been done in Tanzania, as our results reflect, but needs improvement and higher priority according to stakeholders interviewed in this study. Our study identifies unexploited opportunities of integration in other healthcare programmes, as well as an underutilization of HPV machines.

Additionally, a qualitative study among 31 imprisoned women from Malawi showed that even though the women had knowledge about the disease, risk factors, and screening, other measures were also necessary to improve attendance, such as wide availability of screening and a need for female health practitioners to ease discomfort [35]. This is in line with a qualitative study from West Cameroon, which mentioned low accessibility to the programme in terms of cost and distance, and disrespectful treatment by healthcare workers as the largest barriers [36]. In our study, the screening clients were not as concerned with the gender and behaviour of healthcare workers but were equally challenged by time cost and distance to the clinic, as well as exposure during gynaecological exams. We found that this problem could actually be solved by the possibility of HPV self-sampling as the screening clients in our study found HPV self-sampling comfortable and preferred this method over gynaecological screening with VIA. This is in line with other studies from sub-Saharan Africa, including those included in the CONCEPT project [15,17]. One of these studies – a qualitative study among 21 women from Dar es Salaam – showed that women were not comfortable with performing HPV self-sampling without the presence of a nurse [15]. In our study, women and healthcare providers also agreed that self-sampling shows most potential in a facility-based setting, eliminating the advantage of home-testing, which therefore cannot solve the problem of long distances to test facilities.

A mixed-methods study from Burkina Faso concerning the implementation of HPV-based screening showed that HPV-based screening in primary healthcare centres is possible with high numbers of women completing all screening procedures and high satisfactory among the women [37]. However, the study recommends that the waiting time for results needs to be reduced to a single-visit approach to further improve screening uptake. This is in line with what we found in our study.

### Strength and limitations

To the best of our knowledge, this study is the first of its kind in sub-Saharan Africa that investigates barriers and facilitators for implementing WHO’s guideline in the form of primary HPV-based screening by use of the Social Ecological model as theoretical framework. Numerous interviews were conducted with screening clients, healthcare providers, and key stakeholders, which allowed us to gather individual patient perspectives as well as perspectives on issues arising at community and health system levels in relation to implementing HPV-based screening in Tanzania. The healthcare providers and stakeholders were specialists on the subject, and some of the most knowledgeable experts in the country. However, a limitation in this study may be that several healthcare providers and stakeholders were partners in the CONCEPT project, which could introduce a conflict of interest and them being positively biased towards HPV-based screening. This is considered to be a minor issue, as the CONCEPT study is a research project with no financial incentives for implementing HPV-based screening. Further, our findings concerning the screening client’s attitude towards the two tests could have been affected, since we during the interview did a brief health education section about HPV and the two test modalities. Though the education was not biased towards any of the two tests, information in itself could have changed the women’s perspective, which actually supports our finding with need of more health education. The screening clients all attended clinic-based cervical cancer screening and therefore had the resources to participate in screening and reflect upon it. Several of the women were living with HIV, which potentially means that they have more experience with the health system and screening in general, thus making them less inclined to misconceptions regarding cervical cancer screening. As this is a qualitative study that reflects only the views of 16 interviewed screening clients, we cannot draw general conclusions. However, when comparing our findings with other studies conducted in other populations, we identified somewhat similar findings. Finally, this study was the translator’s first experience with ad hoc English-Kiswahili translation, and even though she was fluent in English, she was not a trained translator, so nuances might be missing in the translation.

### Implications for practice

To approach this aim further, a thorough investigation into actual and opportunity costs is needed by policymakers before successful implementation of HPV-based screening in the country can be accomplished. Further, there is a great need for point of care HPV testing, which will be important for the women, eliminate loss of follow-up, and enable screen, triage, and treat in one visit. The lack of knowledge about cervical cancer calls for a national reinforcement by the government, with widespread campaigning on social media and outreach campaigns. Most of our screening participants had been referred through other health services, yet this study shows that this process is not fully exploited as more horizontal integration into existing programmes and technologies used in TB and HIV control could be beneficial. Finally, this study shows a need for a steady supply chain of tests, reactants, and machines in the country, preferably with a local distributor, in order to provide reliable HPV-based screening.

## Conclusions

There are several barriers and facilitators at all societal levels, which Tanzania needs to address in order to successfully implement WHO’s guideline of primary HPV-based screening. HPV-based cervical cancer screening is well perceived and has great potential in the country. However, unavailability of clinic-based screening with a one-visit approach and overcoming of fear and misconceptions are very important struggles that need to be addressed at community and health system level in order to overcome the screening clients’ largest barriers. A lack of a local and steady supply chain and underutilisation of HPV machines and horizontal integration in existing health programmes are barriers that need to be addressed at the political level in order for the guideline to be successfully implemented in Tanzania.

## Supplementary Material

Supplementary file 4 COREQ checklist.docx

Supplementary file 3 Codebook Revised.docx

Supplementary file 2 Interview guide healthcare professionals.docx

Supplementary file 1 Interview guide screening clients.docx

## Data Availability

The dataset analysed in the study can be obtained from the corresponding author upon reasonable request.

## List of abbreviations

WHO: World Health Organization
HPV: Human Papillomavirus
LMIC: Low- and middle-income countries
HDI: Human development index
ASIR: Age-standardized incidence rate
HR: High-risk
HIC: High-income countries
WLWH: Women living with HIV
VIA: Visual inspection with acetic acid
LEEP: Loop electrosurgical excision procedure
CONCEPT: Comprehensive Cervical Cancer Prevention in Tanzania
ORCI: Ocean Road Cancer Institute
KCMC: Kilimanjaro Christian Medical Centre
SDU: University of Southern Denmark
HIV: Human immunodeficiency virus
CTC: HIV care and treatment clinics
TB: Tuberculosis
DANIDA: The Danish International Development Agency
