# Supplementary material for: Barriers and facilitators for implementation of HPV-based cervical cancer screening in Tanzania: a qualitative study among healthcare providers, stakeholders, and Tanzanian women
Source: Glob Health Action. 2025 Apr 24;18(1):2491852. doi: 10.1080/16549716.2025.2491852 (PMC12024489; doi:10.1080/16549716.2025.2491852)
Supplement: Supplementary file 3 Codebook Revised.docx [file ZGHA_A_2491852_SM6139.docx]

**Supplementary file 3:** Codebook

Codebook for analysis of interviews with screening clients, healthcare providers and stakeholders on cervical cancer screening
