# Supplementary material for: Barriers and facilitators for implementation of HPV-based cervical cancer screening in Tanzania: a qualitative study among healthcare providers, stakeholders, and Tanzanian women
Source: Glob Health Action. 2025 Apr 24;18(1):2491852. doi: 10.1080/16549716.2025.2491852 (PMC12024489; doi:10.1080/16549716.2025.2491852)
Supplement: Supplementary file 2 Interview guide healthcare professionals.docx [file ZGHA_A_2491852_SM6138.docx]

**Supplementary file 2:** Interview guide healthcare professionals

Final interview guide for interviews conducted with healthcare professionals and stakeholders

| Topic | Questions |
| --- | --- |
| **Introduction** | *At first, I would like to know something about your background.*   1. What is your educational background? 2. What is your current profession? 3. How long have you been working on KCMC/ORCI? 4. How long have you worked with the topic of cervical cancer and/or screening for cervical cancer? 5. The largest burden of cervical cancer is found in East Africa – why do you think that is? |
| **Key questions**  The current screening programme with VIA | *The next questions will revolve the current screening programme in Tanzania.*   1. Can you describe the Tanzanian cervical cancer screening programme for me?    1. Are women attending the screening programme? Why/why not? 2. Can you explain to me what screening methods that are currently used in the screening programme in Tanzania? 3. What general challenges do you see with the current screening programme?    1. Are there any expenses linked to the programme? 4. How are women who screen positive followed up? (Any challenges with lost to follow-up?) 5. Can you describe the typical woman, who is using the screening programme?    1. When does a woman come for screening? Routine check? After symptoms? Something else? 6. What is the general attitude towards the screening programme? Is it helping? Hopeful? Unnecessary?    1. Among the population/women?    2. Among healthcare providers? |
| **Key questions**  The updated guidelines from WHO | *The next questions will be about WHO’s 2021 guidelines regarding cervical cancer screening.*   1. Have you been informed on WHO’s recommended guidelines for cervical cancer prevention from 2021?   *Description of guidelines: In brief they suggest that all women over 30 must have performed an HPV DNA detection in either a screen-and-treat or a screen, triage and treat approach every 5 to 10 years. If the women are living with HIV the HPV DNA detection needs to be performed every 3 to 5 years starting at the age of 25 years in a screen, triage and treat approach.*   1. What is your opinion about the new guidelines?    1. Is it feasible to follow this guideline in Tanzania? (Why/why not?)    2. What do you think about the age-limits, the screening-interval, the distinguish between HIV-status? 2. In your opinion, what are the pros and cons with using HPV-based screening compared to VIA?    1. Challenges with follow-up do to waiting time for results?    2. Sensitivity?    3. The possibility for self-sampling? 3. Have you ever worked with HPV-screening in the clinic? What was your thoughts about it?    1. How did you follow-up the women who screened positive? |
| **Key questions**  Economy | *In the next questions, I would like your opinion about different potential challenges regarding use of HPV-testing as primary screening method.*   1. Which economic costs will there be, when changing from VIA to HPV self-sample? 2. From an economic perspective, is it possible to implement HPV-based primary screening in the Tanzanian screening programme?    1. If yes, why?    2. If no, what would it take? |
| **Key questions**  Execution and practicalities | *Screening by use of HPV-testing requires more equipment and laboratory facilities than VIA.*   1. Do you think that the healthcare system has the necessary equipment to switch to HPV-based screening? (Why/why not?) (Equipment shortage? Other organizational issues?) 2. Is it possible to conduct “screen-and-treat” in one visit with HPV-testing, similar to VIA? (Why/why not?)    1. If HPV tests could deliver the result on the same day as the test was taken, how do you think that would affect the feasibility of using HPV-testing in Tanzania? |
| **Key questions**  Personnel | 1. According to your point of view, will HPV-based screening require another level healthcare providers than VIA-based screening? (Enough trained gynaecologists, nurses, pathologists etc.?) 2. Do you think it is possible to incorporate the new screening procedure in the Tanzanian healthcare system based on a personnel perspective?    1. If yes, why?    2. If no, what would it take? |
| **Closing** | *Thank you for your time and your expertise.*   1. Is there anything else you would like to add? |
