# Supplementary material for: Barriers and facilitators for implementation of HPV-based cervical cancer screening in Tanzania: a qualitative study among healthcare providers, stakeholders, and Tanzanian women
Source: Glob Health Action. 2025 Apr 24;18(1):2491852. doi: 10.1080/16549716.2025.2491852 (PMC12024489; doi:10.1080/16549716.2025.2491852)
Supplement: Supplementary file 1 Interview guide screening clients.docx [file ZGHA_A_2491852_SM6137.docx]

**Supplementary file 1:** Interview guide screening clients

Final interview guide for interviews conducted with screening clients

| Topic | Questions |
| --- | --- |
| **A. Introduction**  Today’s screening experience | *Thank you for coming today and for participating is this interview. It is a great help to us. We are very grateful.*  *So, you have just been to the clinic.*   1. Can you tell me why you are here today?    1. Why do you think the nurses and the doctor wants to see you again? 2. Can you tell me what you have just experienced? 3. Do you know what kind of procedure, that was done and why?    1. Do you know any other screening methods for cervical cancer? 4. How did you feel about the examination? (Good? Safe? Did it hurt? Was it uncomfortable?) 5. Is there anything today that would have made the examination more comfortable for you? 6. Can I ask, how old are you? 7. Have you experienced any symptoms since your last screening – i.e., bleeding outside of your period or after menopause, change in your period, bleeding during intercourse, change in discharge, pelvic pain? |
| **B. Key questions**  Screening with HPV self-sample test | *I will show you this test* (show the woman the Evalyn Brush).   1. Have you previously tried this test yourself or accompanied by a nurse or doctor? 2. If yes, how did it feel? (Good? Safe? Did it hurt? Was it uncomfortable?) 3. Did you do the test at your own home?    1. If yes, did you like this? 4. What did you like about the HPV self-sample test? 5. What did you not like about the HPV self-sample test? 6. How did you get the answer of the test?    1. Did you like this way of getting the results? 7. Do you know what it means to HPV positive? |
| **C. Key questions**  Screening with VIA | *Now I would like to ask you questions about earlier examinations. You can maybe think of the last time you had a cervical cancer screening.*   1. Can you explain to me the last time you had an examination at the clinic?    1. Do you know which kind of examination you had?    2. How did it feel? (Good? Safe? Did it hurt? Was it uncomfortable?) 2. What did you like about the examination? 3. What did you not like about it? 4. How did you get the answer of the examination?    1. Did you like this way of getting the results? |
| **D. Key questions**  Comparison of VIA and HPV-DNA test | *If you have tried both the regular cervical cancer screening (with VIA) and the HPV- test* (show the Evalyn Brush)*, I would like to know how you feel about them compared to each other.*   1. How did you like the test, compared to a regular gynaecological examination? Why? 2. Would you prefer screening in your own home or at the clinic? Why? 3. Would you prefer the answer right away or is it ok to wait? Why?   ***Clarification on screening methods:***  *The spray (VIA) can show lesions on the cervix, that can be a sign of early cancer. You can have the answer right away during the examination. You need a gynaecological exam at a clinic, and the spray might itch or burn. If you have lesions, you can be treated right away. The spray is not very sensitive, and not all lesions will be detected, and sometimes lesions are first discovered in a late stage.*  *The HPV-test will show you if you have HPV or not. If you are negative, you won’t need any further examination and are in no risk for CC. If you are positive, you should come to the clinic to have a gynaecological exam with either a biopsy or a smear, to see if you have any lesions. After this you will need to wait on the results, before receiving treatment if necessary.  The HPV-test can be done by yourself after instructions, and it can be done in your own home, as you won’t need a gynaecological examination. You must wait on the HPV-results. The test is very sensitive, and if you are negative, you don’t need any other examination.*   1. After receiving this information, have your answers changed? |
| **E. Key questions**  The general screening program | *It is recommended that all women from the age of 30 to be screened for cervical cancer every 5 to 10 years, and even more if you are living with HIV.*   1. How does the general screening work here in Tanzania? 2. How often have you attended screening?    1. Why did you attend screening? 3. We know a lot of women do not attend screening – why do you think that is? 4. How do you think more women would attend? |
| **F. Closing** | *Thank you for your time and for all your answers. Before we finish:*   1. Is there anything else you would like to add? |
